# Supplementary figures and images for: Naturally occurring mutations in replication proteins of a small RNA virus that alter the number, sizes, and relative abundances of subgenomic RNAs
Source: PLoS Pathog. 2026 Jul 7;22(7):e1013842. doi: 10.1371/journal.ppat.1013842 (PMC13340797; doi:10.1371/journal.ppat.1013842)

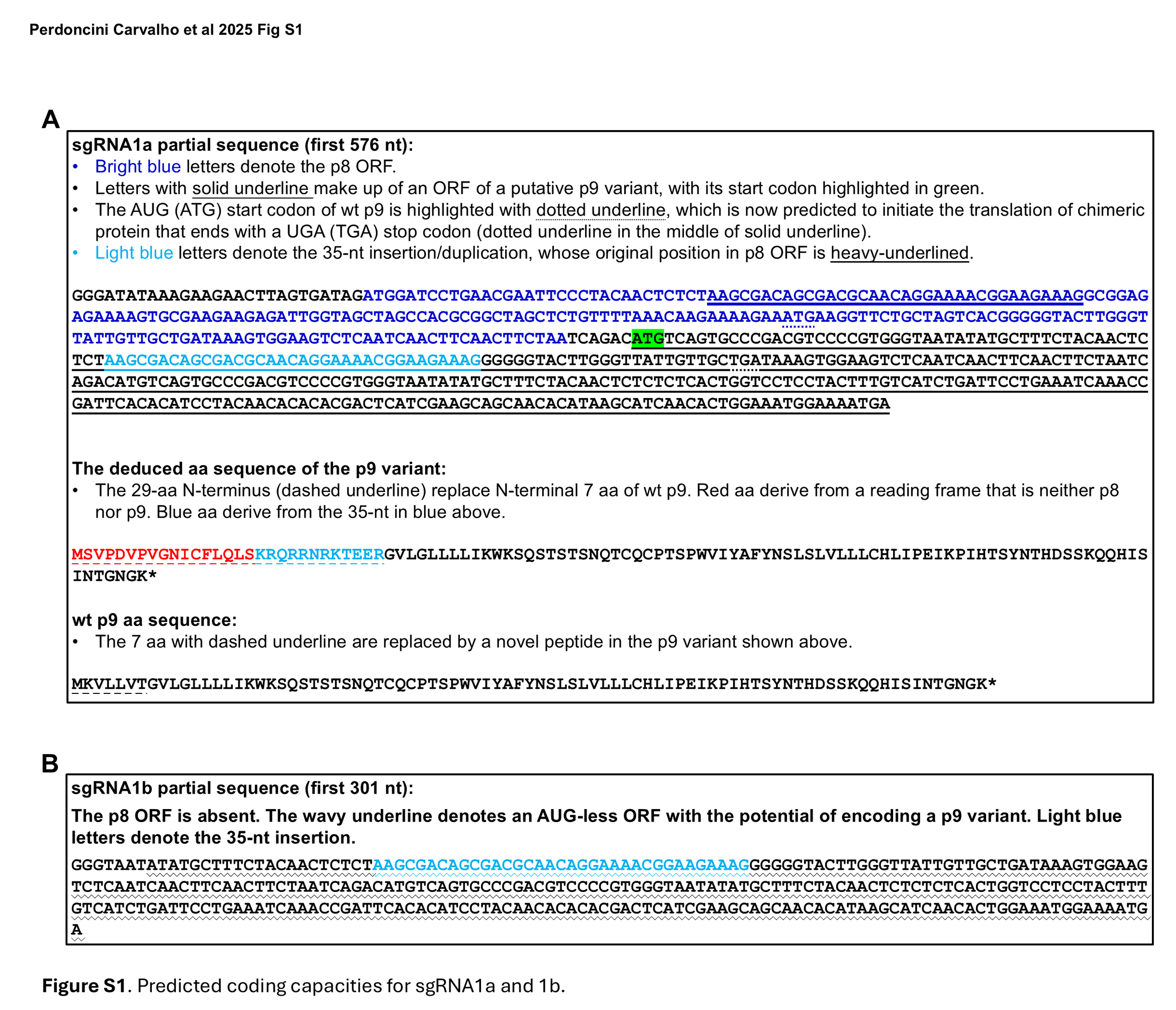

Supplement: S1 Fig — (TIF) [file ppat.1013842.s001.tif]

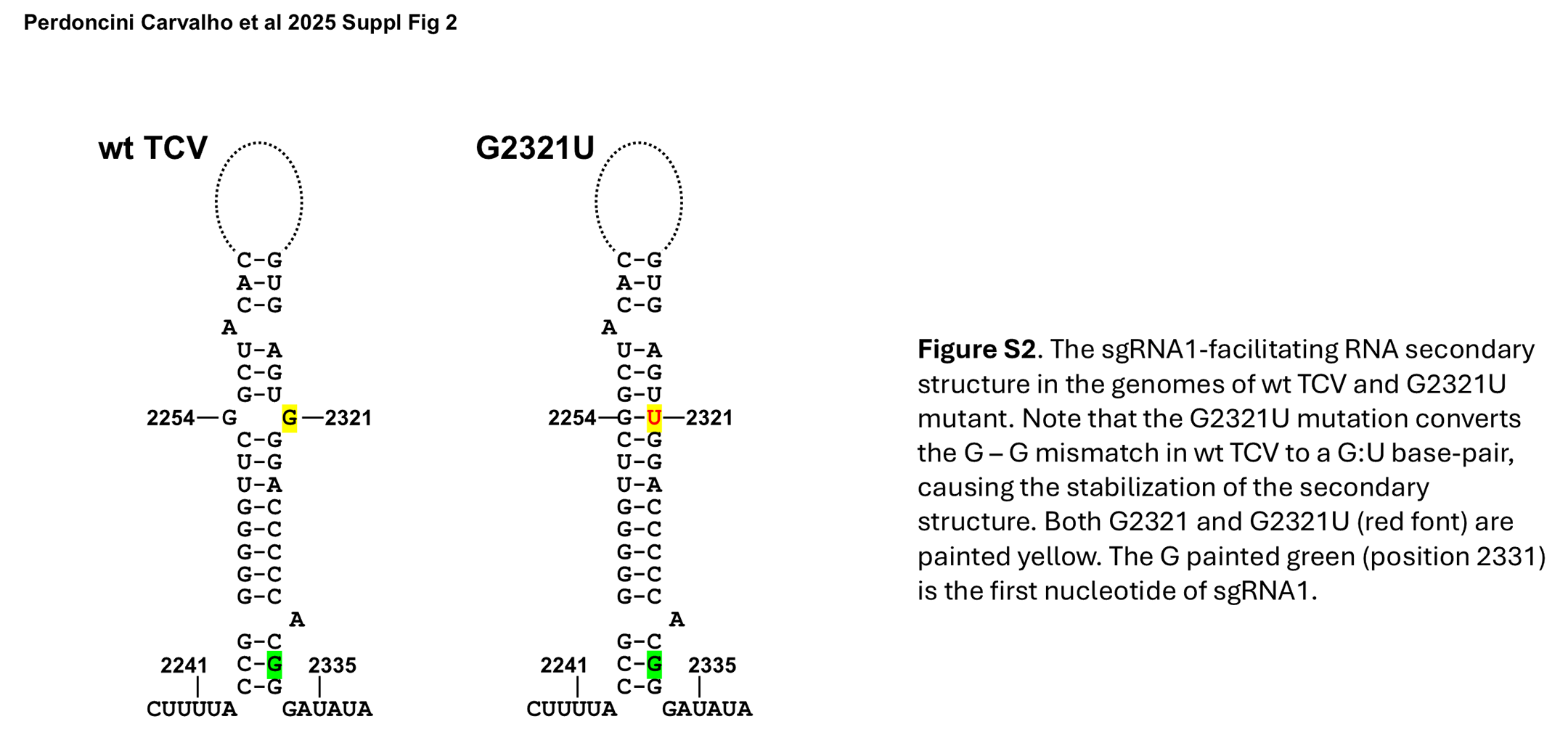

Supplement: S2 Fig — Note that the G2321U mutation converts the G – G mismatch in wt TCV to a G:U base-pair, causing the stabilization of the secondary structure. Both G2321 and G2321U (red font) are painted yellow. The G painted green (position 2331) is the first nucleotide of sgRNA1. (TIF) [file ppat.1013842.s002.tif]
