# Supplementary material for: Naturally occurring mutations in replication proteins of a small RNA virus that alter the number, sizes, and relative abundances of subgenomic RNAs
Source: PLoS Pathog. 2026 Jul 7;22(7):e1013842. doi: 10.1371/journal.ppat.1013842 (PMC13340797; doi:10.1371/journal.ppat.1013842)
Supplement: S1 Raw Image — Each of the raw images contains labels and explanations that connect them to corresponding figure panels. (PDF) [file ppat.1013842.s003.pdf]

Perdoncini Carvalho et al 2026

Original Blot and Gel for Figure 1C

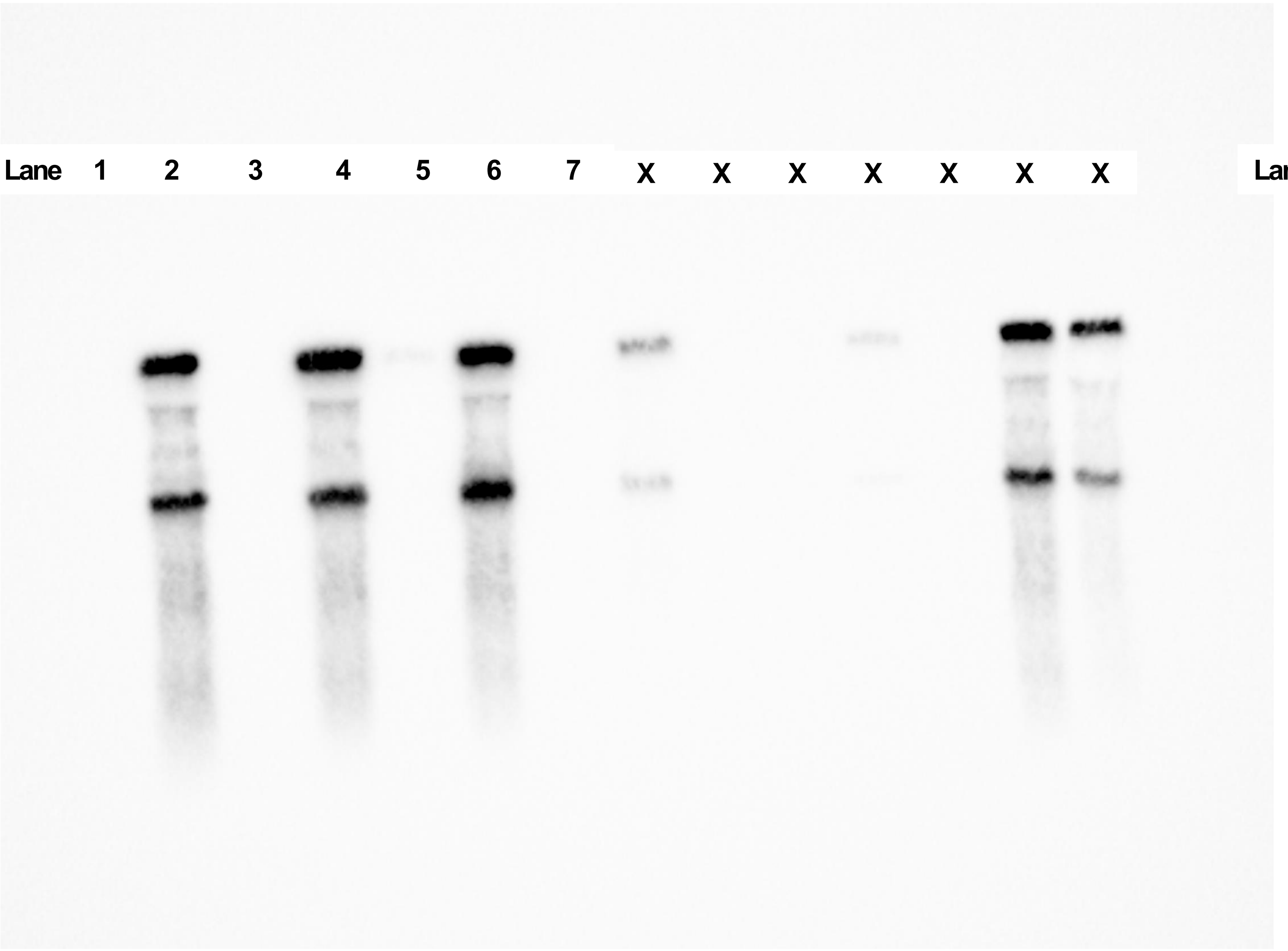

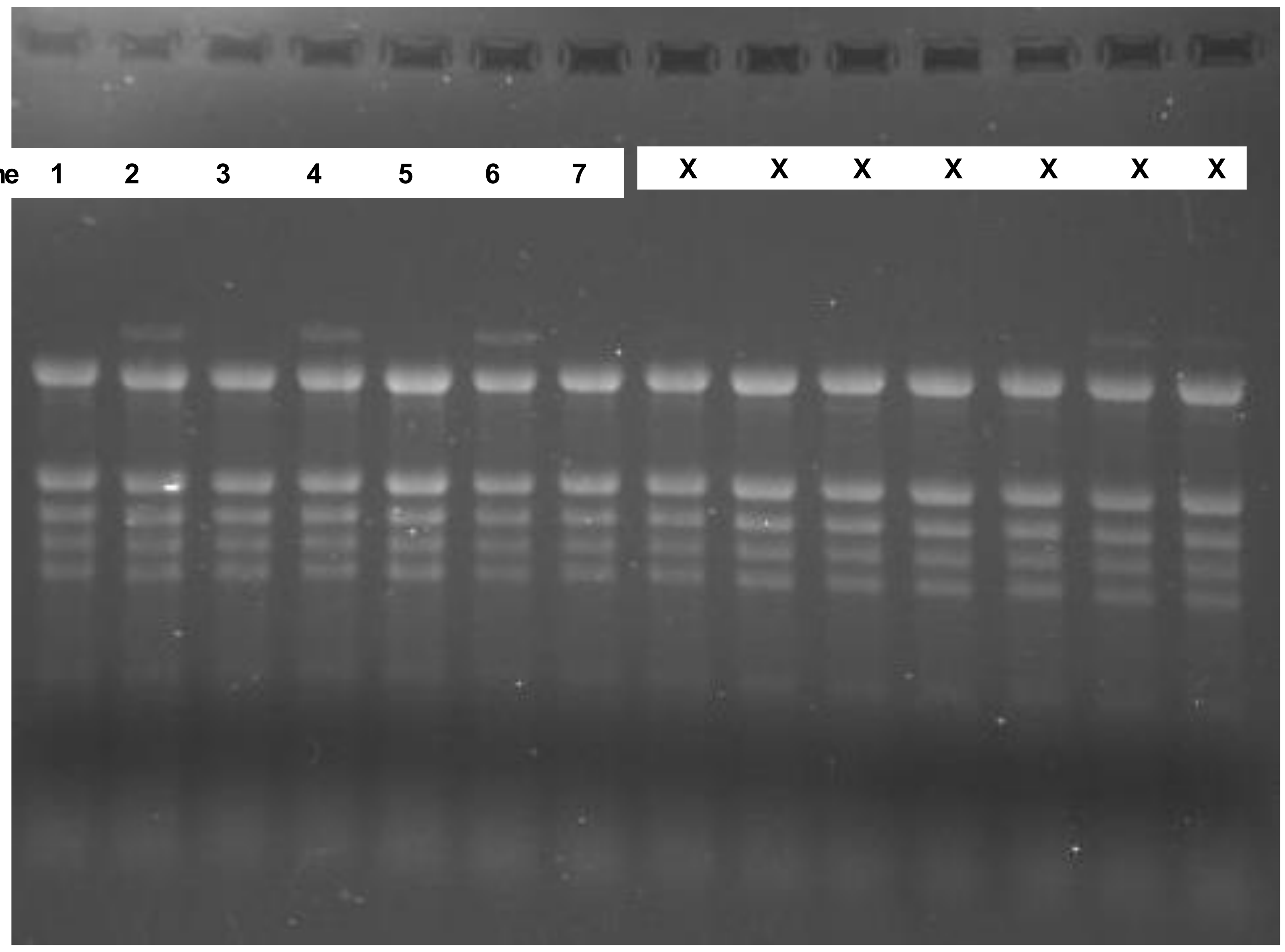

Loading order:
<u>Figure 1C</u>
1: 4dpai IL - P19
2: 4dpai IL - P19 + TCV-dMP_sg2R
3: 4dpai IL - P19 + TCV-dMP-Mut17_sg2R
4: 4dpai IL - P19 + TCV-dMP-A113V_sg2R
5: 4dpai IL - P19 + TCV-dMP-R204H_sg2R
6: 4dpai IL - P19 + TCV-dMP-N303N_sg2R
7: 4dpai IL - P19 + TCV-dMP-G757*_sg2R

Lanes marked with “X” are not part of the Figure. Same for other blots and gel images.

All gel and blot pictures were captured using a ChemiDoc gel imager (BIORAD).
Gels were captured using the Image Lab software Application: Nucleic Acid Gels – Ethidium Bromide
Blots were captured using the Image Lab software Application: Blot - Chemi

Perdoncini Carvalho et al 2026

Original Bot and Gel for Figures 2A and 2B

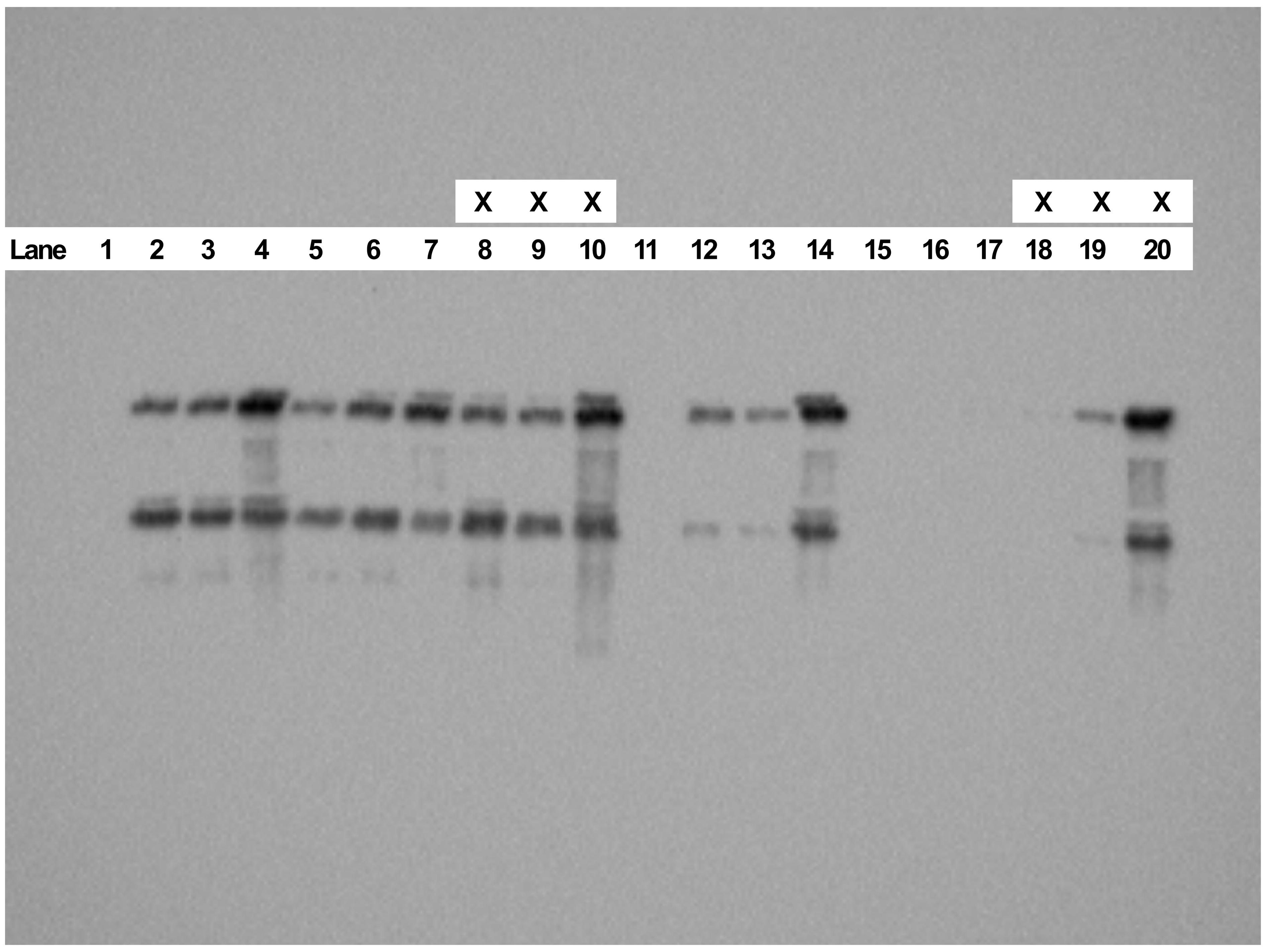

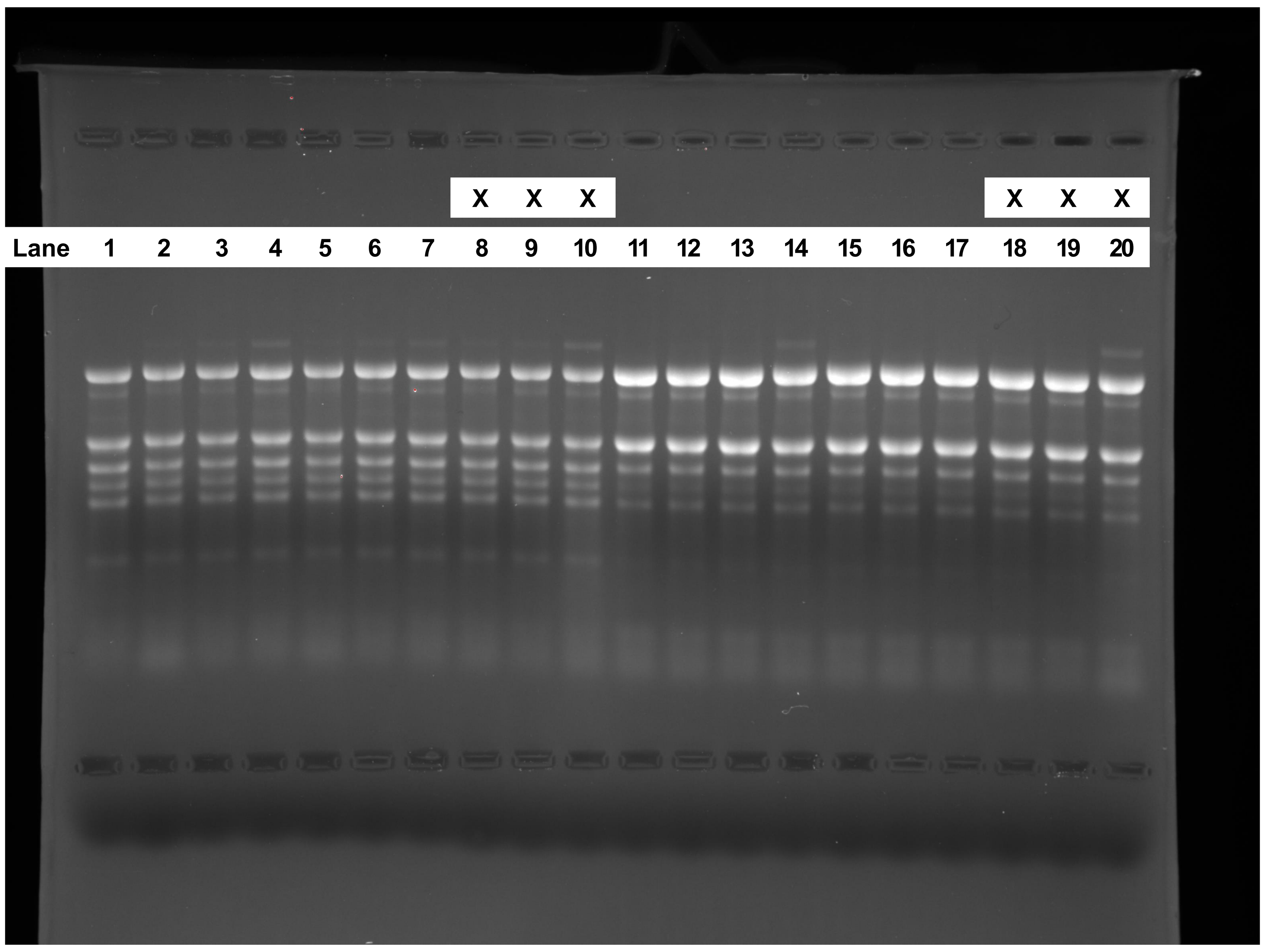

Loading order:

Figure 2A

1: 4dpai IL Mock
2: 4dpai IL wt TCV (OD=1)
3: 4dpai IL wt TCV (OD=0.1)
4: 4dpai IL wt TCV (OD=0.01)
5: 4dpai IL A113V (OD=1)
6: 4dpai IL A113V (OD=0.1)
7: 4dpai IL A113V (OD=0.01)

Figure 2B

11: 7dpai SL Mock
12: 7dpai SL wt TCV (OD=1)
13: 7dpai SL wt TCV (OD=0.1)
14: 7dpai SL wt TCV (OD=0.01)
15: 7dpai SL A113V (OD=1)
16: 7dpai SL A113V (OD=0.1)
17: 7dpai SL A113V (OD=0.01)

Lanes marked with “X” are not part of the Figure. Same for other blots and gel images.

Original Bot and Gel for Figure 2C

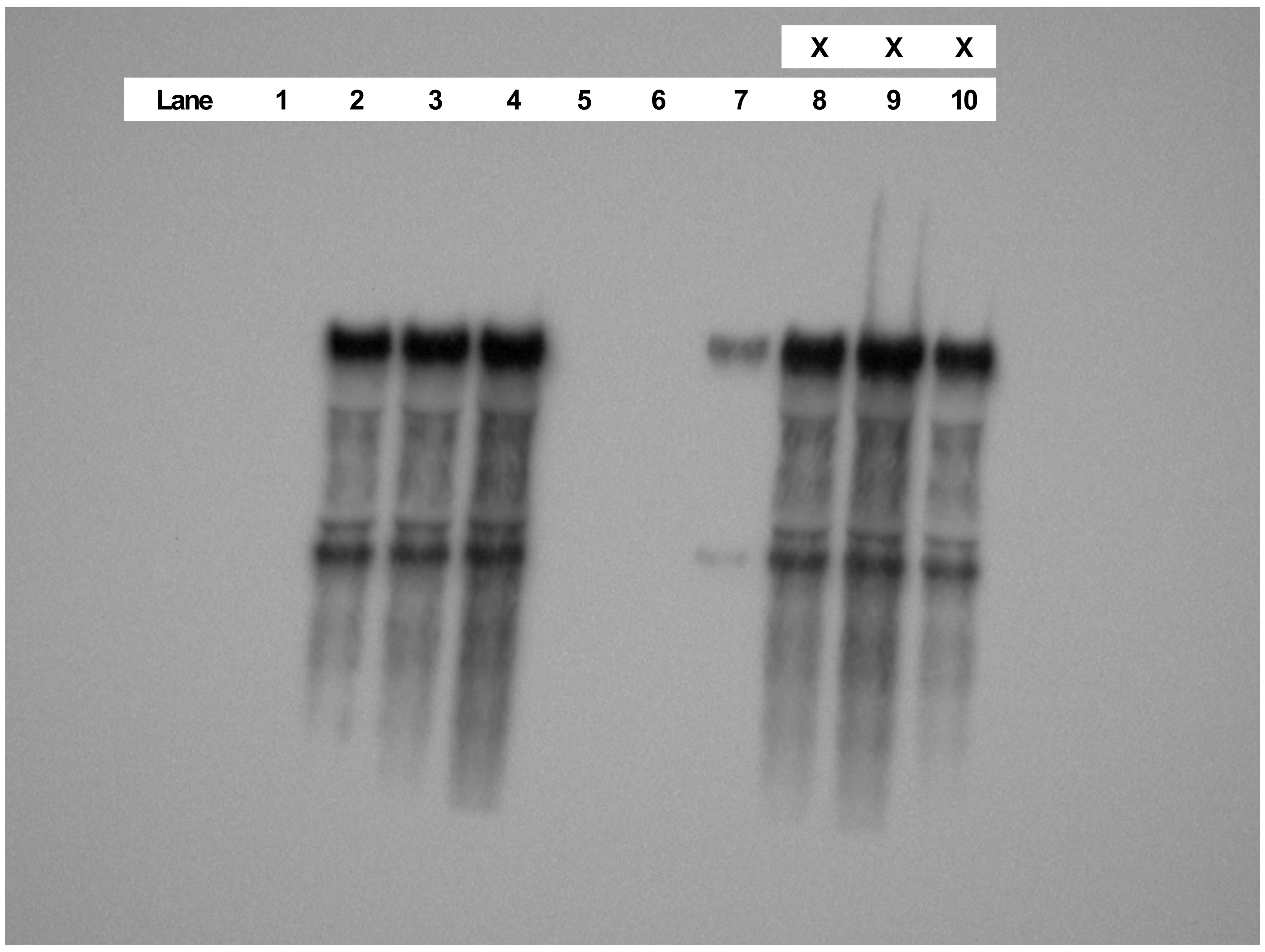

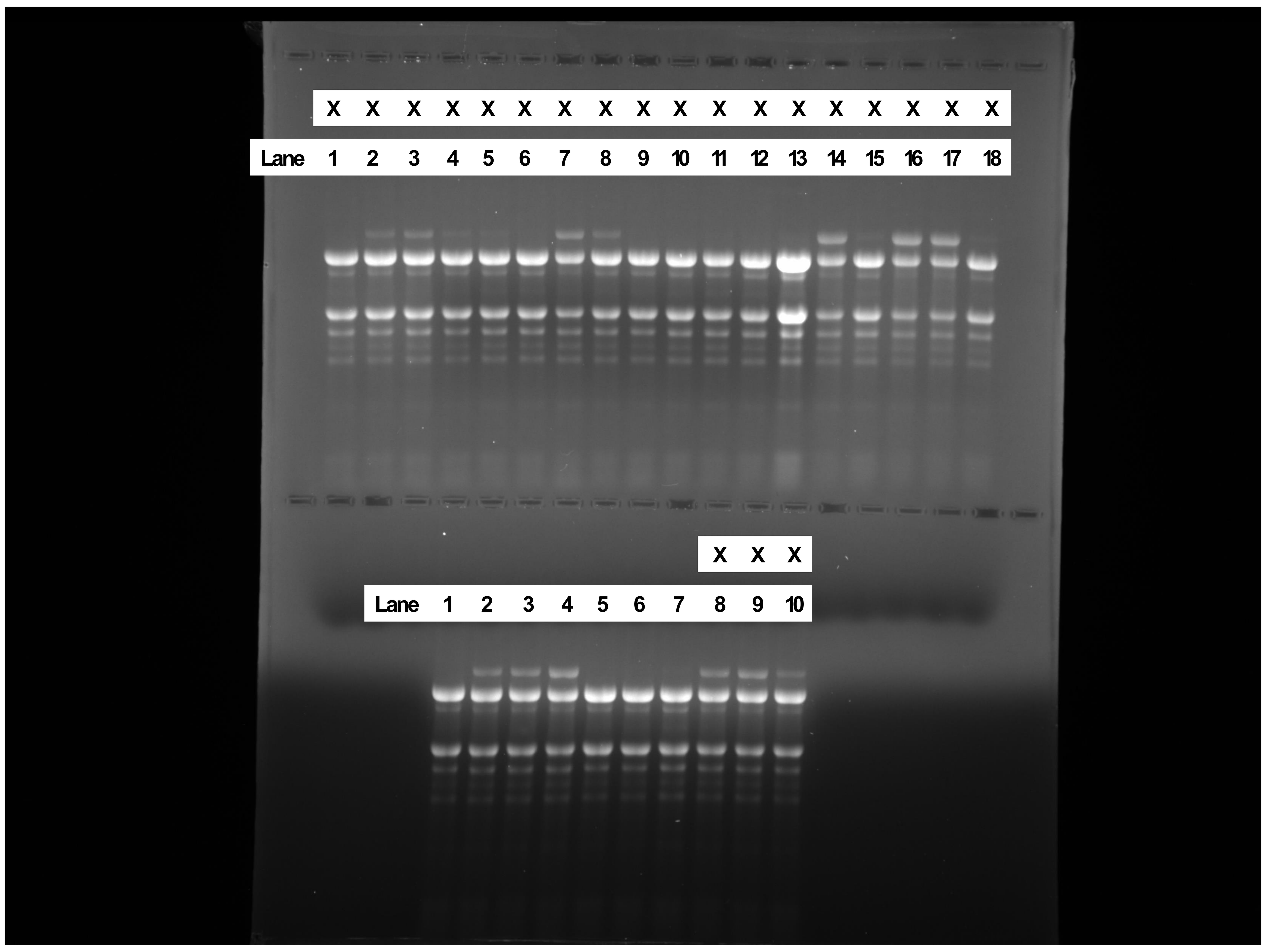

Loading order:

Figure 2C

1: 14dpai SL Mock
2: 14dpai SL wt TCV (OD=1)
3: 14dpai SL wt TCV (OD=0.1)
4: 14dpai SL wt TCV (OD=0.01)
5: 14dpai SL A113V (OD=1)
6: 14dpai SL A113V (OD=0.1)
7: 14dpai SL A113V (OD=0.01)

Lanes marked with “X” are not part of the Figure. Same for other blots and gel images.

Original Bot and Gel for Figure 2D

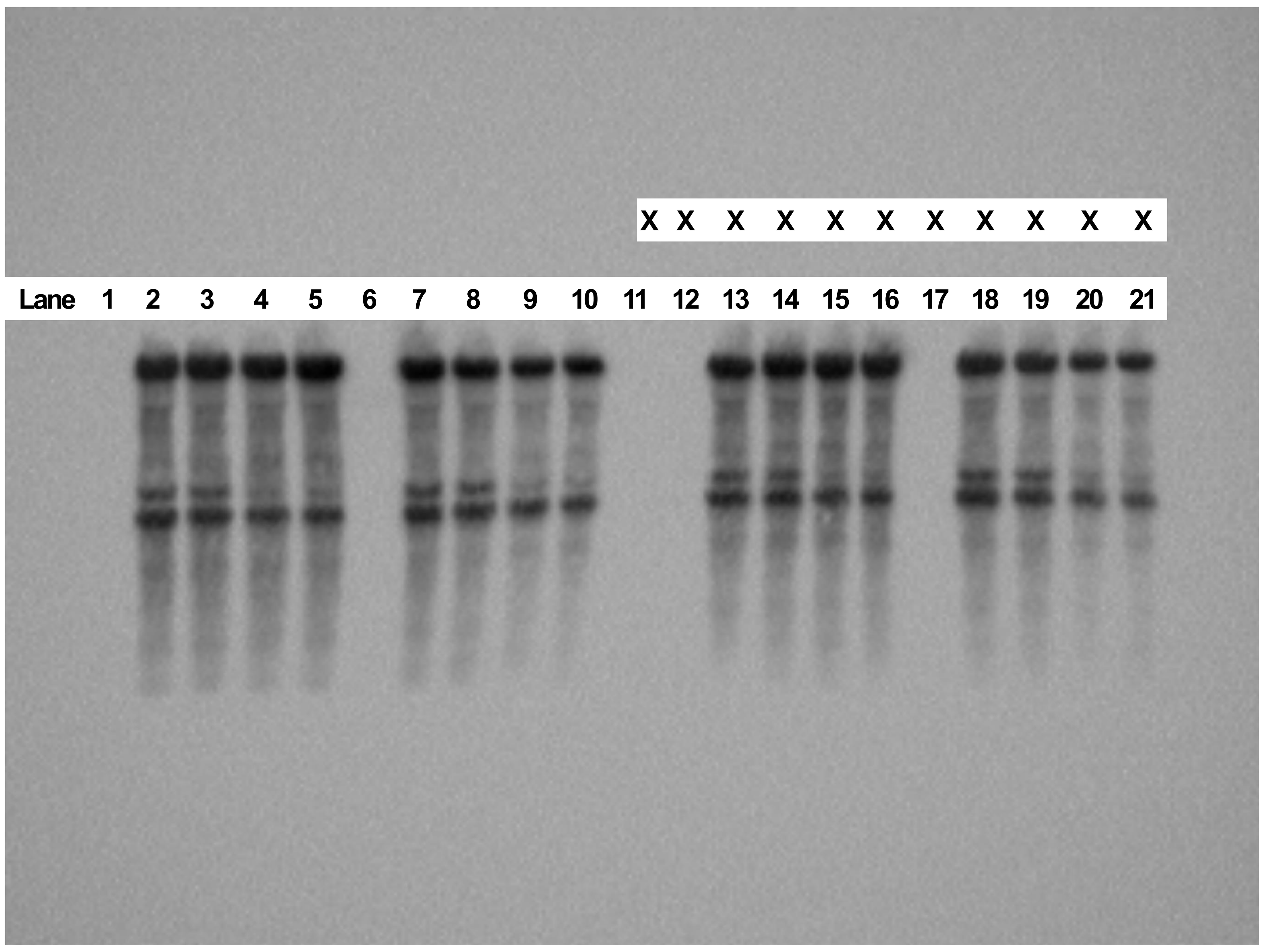

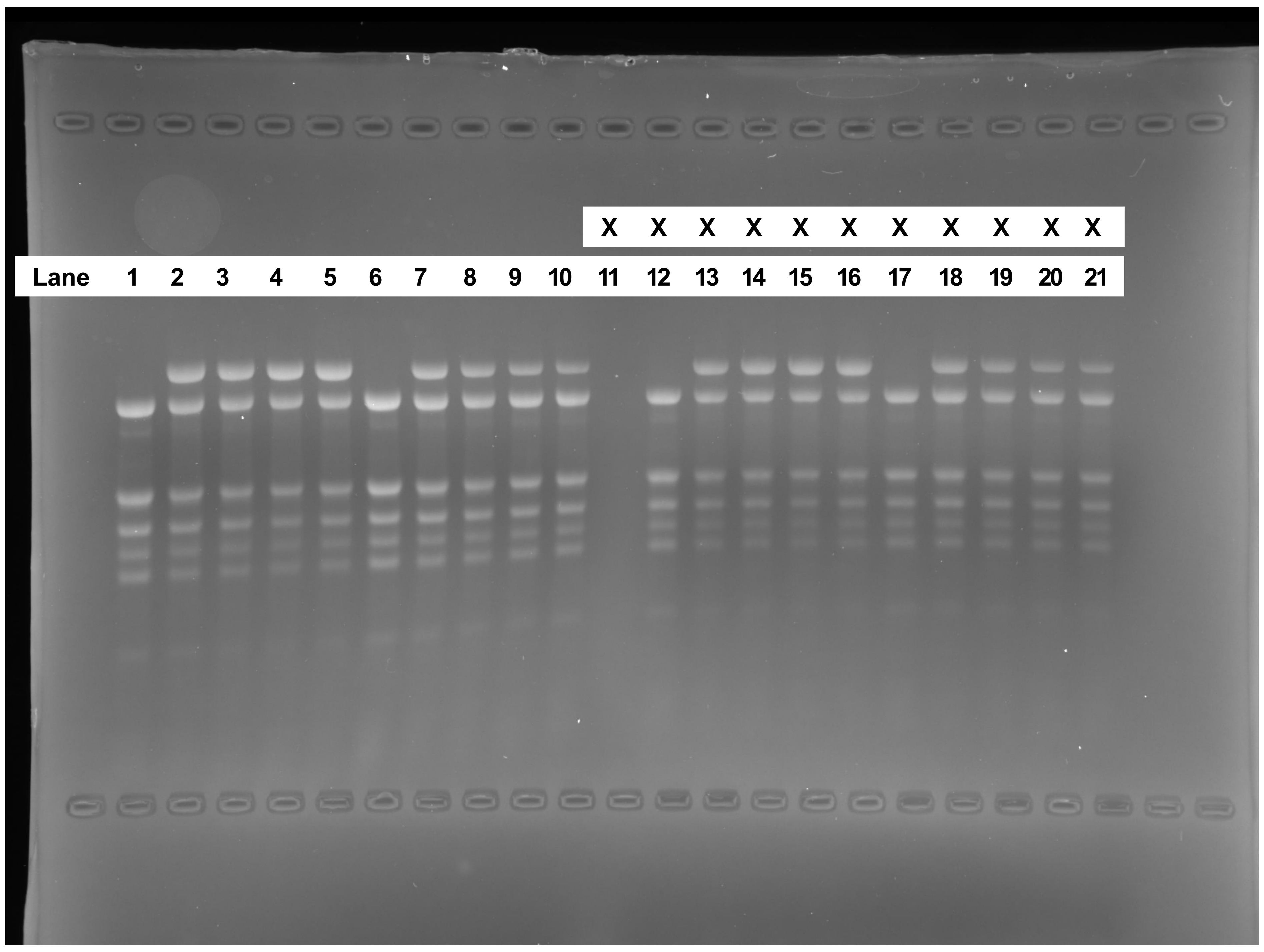

Loading order:
<u>Figure 2D</u>
1: 4dpai IL (MP+) Mock
2: 4dpai IL (MP+) wt TCV
3: 4dpai IL (MP+) wt TCV
4: 4dpai IL (MP+) A113V
5: 4dpai IL (MP+) A113V
6: 4dpai IL (wt N.b.) Mock
7: 4dpai IL (wt N.b.) wt TCV
8: 4dpai IL (wt N.b.) wt TCV
9: 4dpai IL (wt N.b.) A113V
10: 4dpai IL (wt N.b.) A113V

Lanes marked with "X" are not part of the Figure. Same for other blots and gel images.

Original Bot and Gel for Figures 2E and 2F

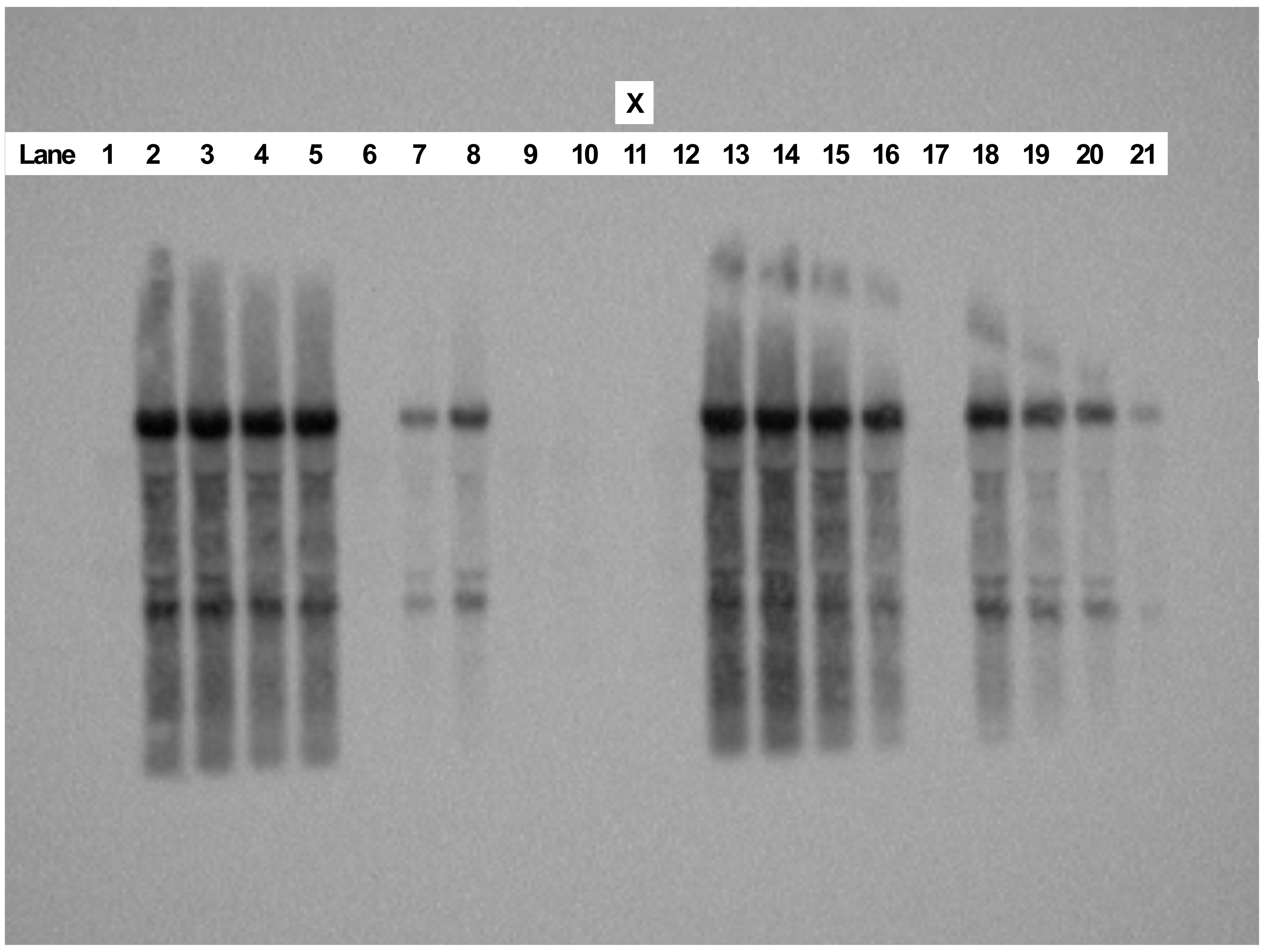

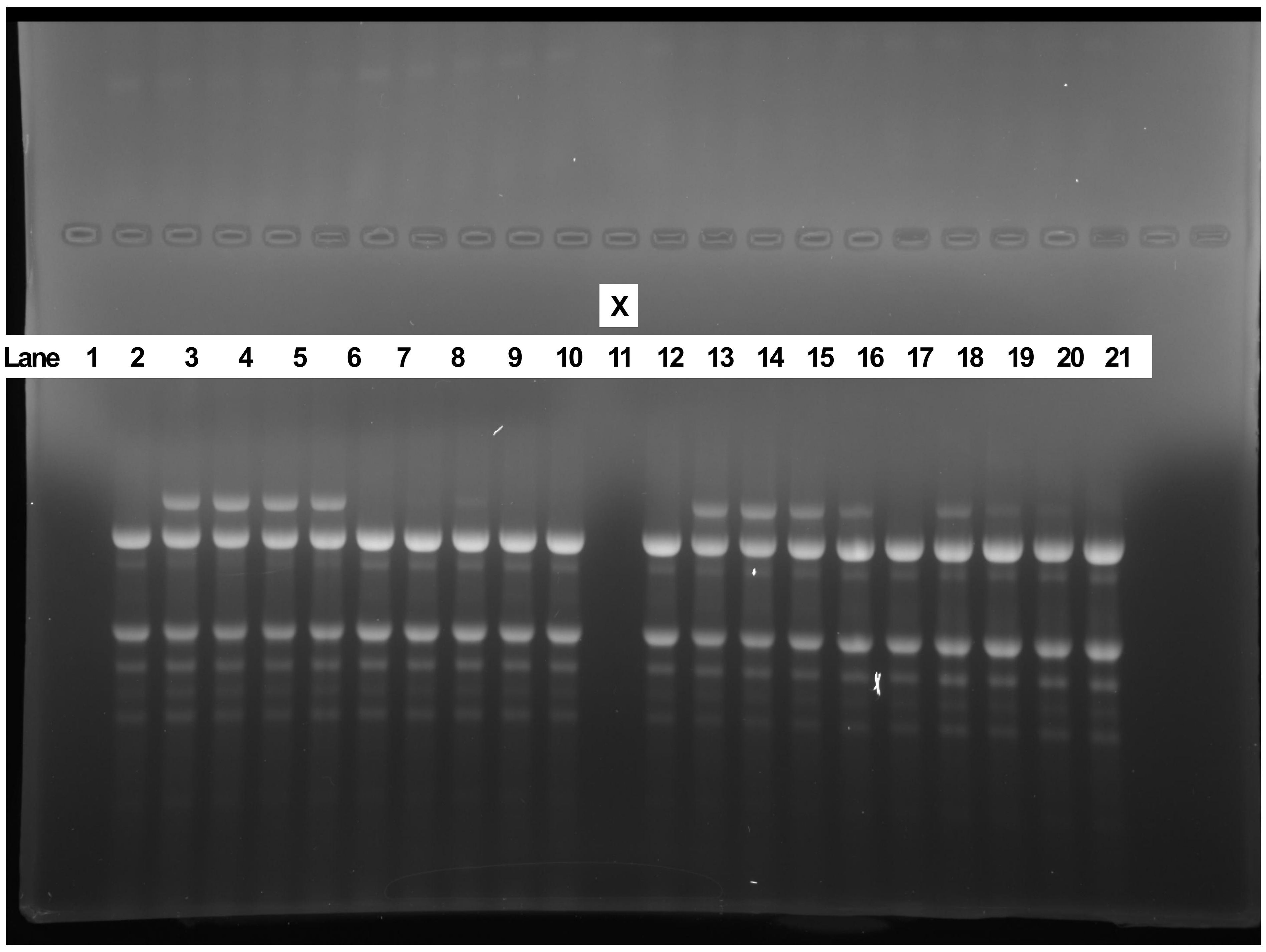

Loading order:

<u>Figure 2E</u>
1: 7dpai SL (MP+) Mock
2: 7dpai SL (MP+) wt TCV
3: 7dpai SL (MP+) wt TCV
4: 7dpai SL (MP+) A113V
5: 7dpai SL (MP+) A113V
6: 7dpai SL (wt N.b.) Mock
7: 7dpai SL (wt N.b.) wt TCV
8: 7dpai SL (wt N.b.) wt TCV
9: 7dpai SL (wt N.b.) A113V
10: 7dpai SL (wt N.b.) A113V

<u>Figure 2F</u>
12: 14dpai SL (MP+) Mock
13: 14dpai SL (MP+) wt TCV
14: 14dpai SL (MP+) wt TCV
15: 14dpai SL (MP+) A113V
16: 14dpai SL (MP+) A113V
17: 14dpai SL (wt N.b.) Mock
18: 14dpai SL (wt N.b.) wt TCV
19: 14dpai SL (wt N.b.) wt TCV
20: 14dpai SL (wt N.b.) A113V
21: 14dpai SL (wt N.b.) A113V

Lanes marked with "X" are not part of the Figure. Same for other blots and gel images.

Perdoncini Carvalho et al 2026

Original Bot and Gel for Figure 3B

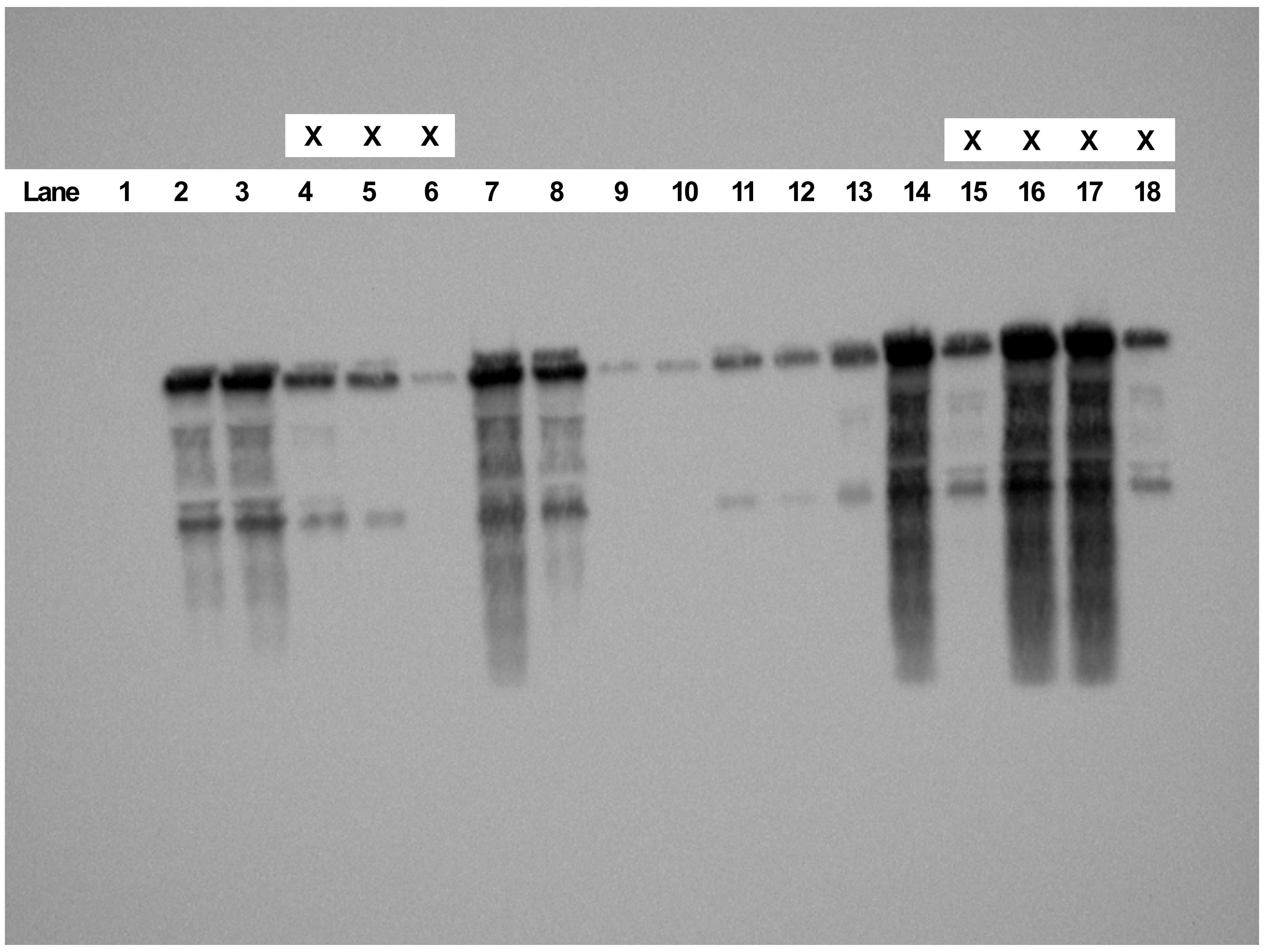

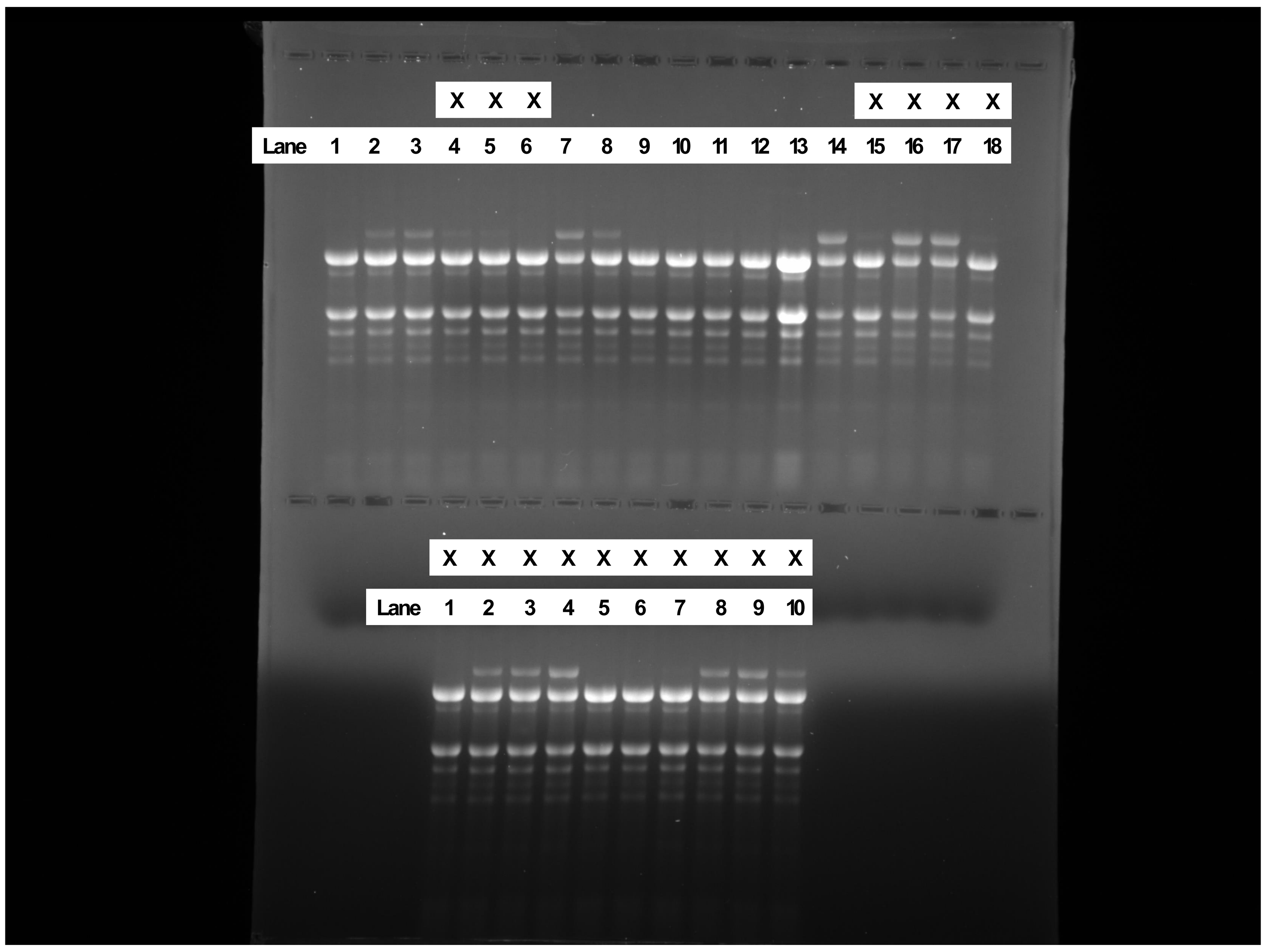

Loading order:

Figure 3B

1: 21dpai SL (wt N.b.) Mock
2: 21dpai SL (wt N.b.) wt TCV (OD=0.1)
3: 21dpai SL (wt N.b.) wt TCV (OD=0.01)

7: 21dpai SL (wt N.b.) A113V (OD=0.1) – Plant 1
8: 21dpai SL (wt N.b.) A113V (OD=0.1) – plant 2
9: 21dpai SL (wt N.b.) A113V (OD=0.1) – Plant 3
10: 21dpai SL (wt N.b.) A113V (OD=0.1) – Extra plant
11: 21dpai SL (wt N.b.) A113V (OD=0.01) – Plant 4
12: 21dpai SL (wt N.b.) A113V (OD=0.01) – Plant 5
13: 21dpai SL (wt N.b.) A113V (OD=0.01) – Plant 6
14: 21dpai SL (wt N.b.) A113V (OD=0.01) – Extra plant

Lanes marked with “X” are not part of the Figure. Same for other blots and gel images.

Original Bot and Gel for Figure 4B

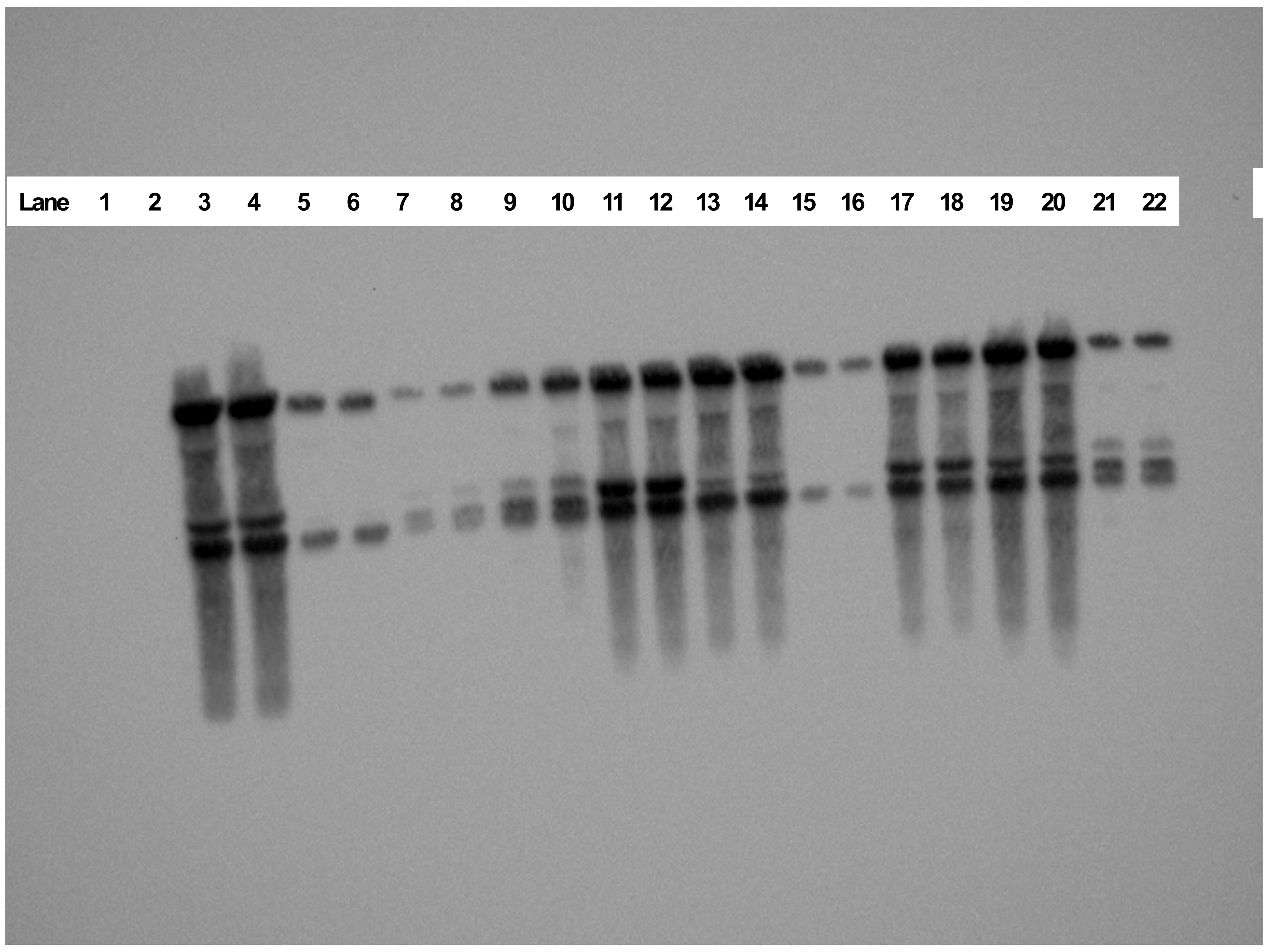

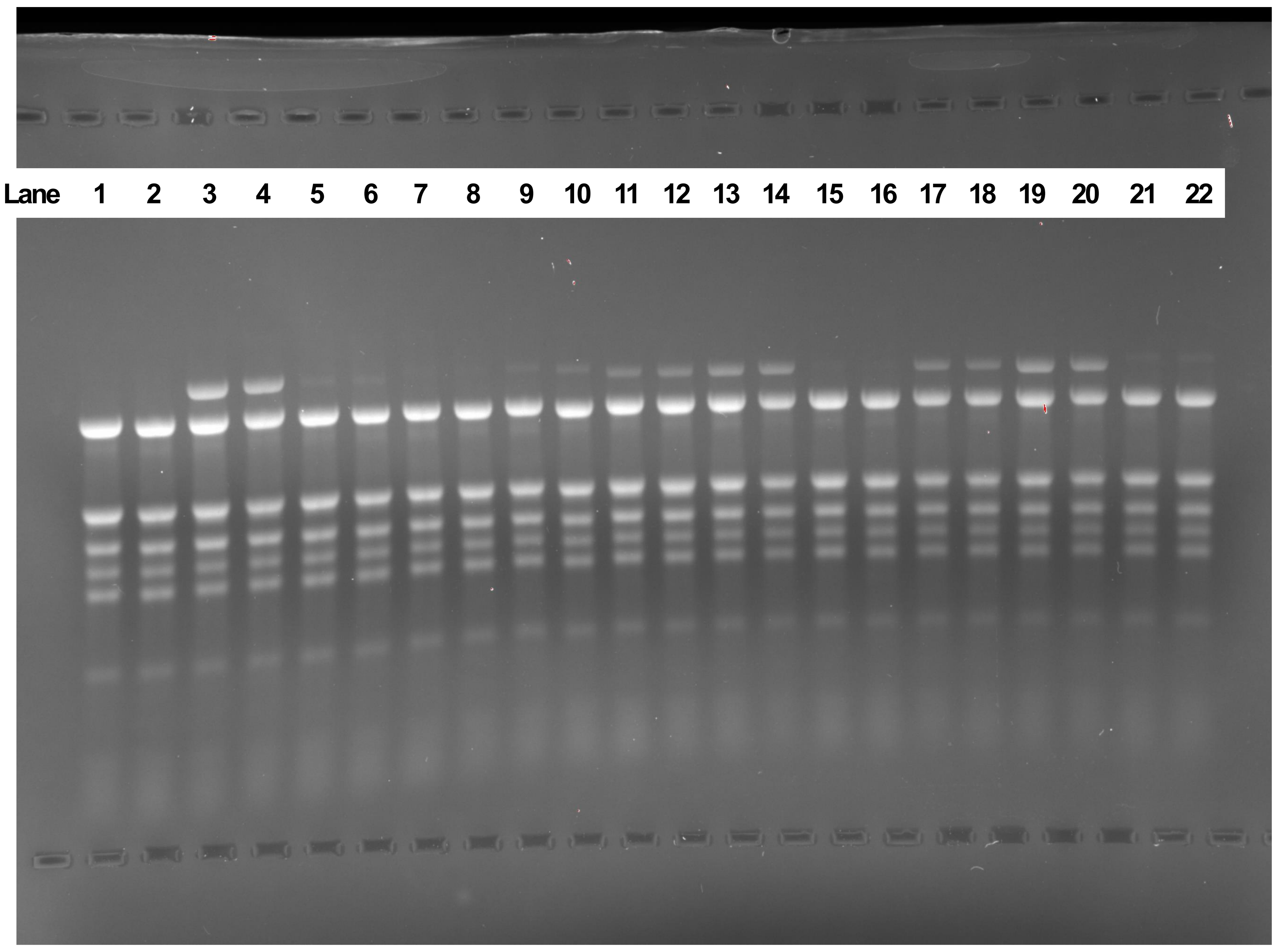

Loading order:
<u>Figure 4B</u>

1: 4dpai IL (wt N.b.) Mock
2: 4dpai IL (wt N.b.) Mock
3: 4dpai IL (wt N.b.) wt TCV
4: 4dpai IL (wt N.b.) wt TCV
5: 4dpai IL (wt N.b.) K389E
6: 4dpai IL (wt N.b.) K389E
7: 4dpai IL (wt N.b.) E392K
8: 4dpai IL (wt N.b.) E392K
9: 4dpai IL (wt N.b.) KEEK
10: 4dpai IL (wt N.b.) KEEK
11: 4dpai IL (wt N.b.) W753L
12: 4dpai IL (wt N.b.) W753L
13: 4dpai IL (wt N.b.) A113V
14: 4dpai IL (wt N.b.) A113V
15: 4dpai IL (wt N.b.) A113V + K389E
16: 4dpai IL (wt N.b.) A113V + K389E
17: 4dpai IL (wt N.b.) A113V + E392K
18: 4dpai IL (wt N.b.) A113V + E392K
19: 4dpai IL (wt N.b.) A113V + KEEK
20: 4dpai IL (wt N.b.) A113V + KEEK
21: 4dpai IL (wt N.b.) A113V + W753L
22: 4dpai IL (wt N.b.) A113V + W753L

Lanes marked with "X" are not part of the Figure. Same for other blots and gel images.

Original Bot and Gel for Figure 4C

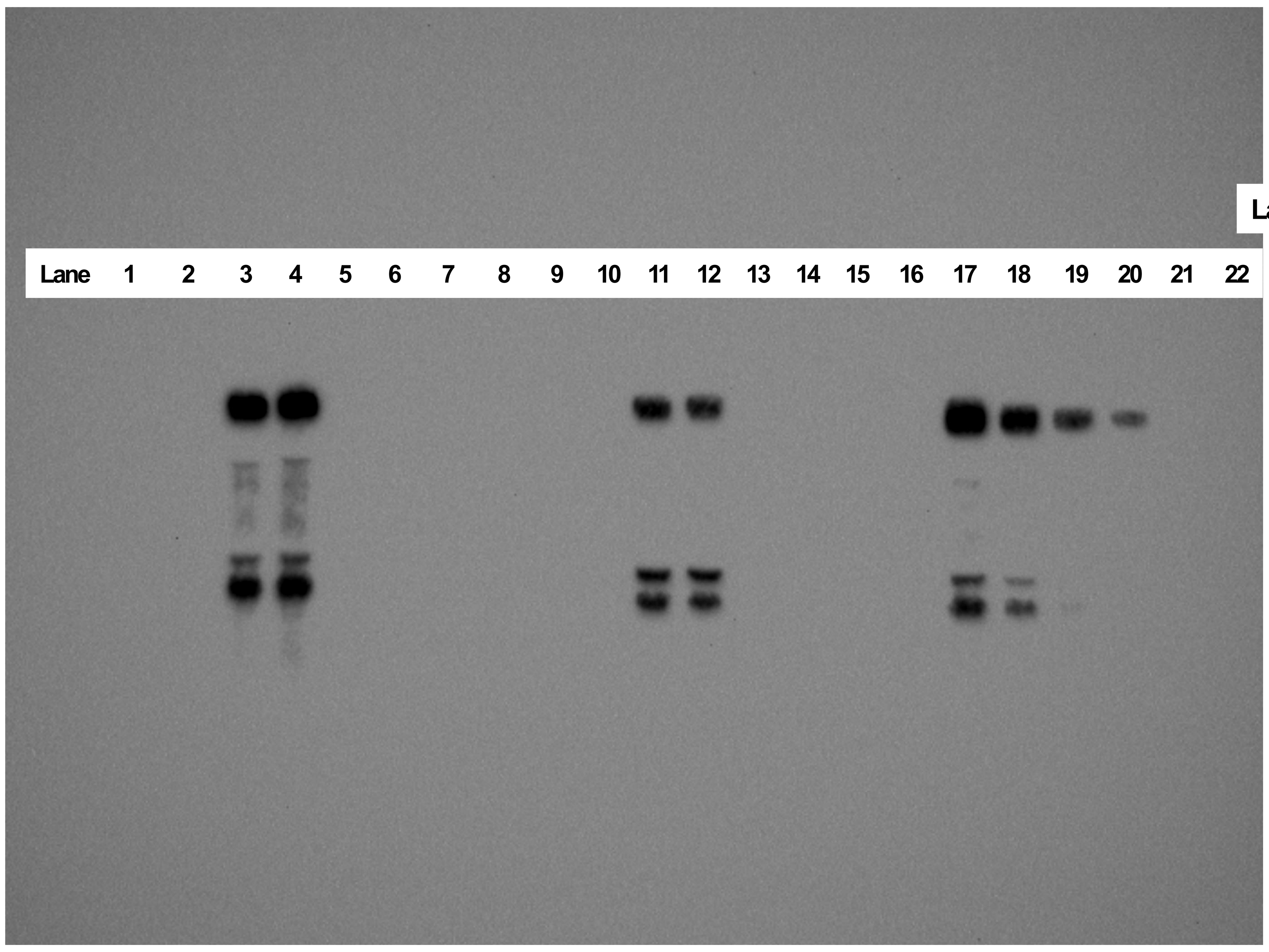

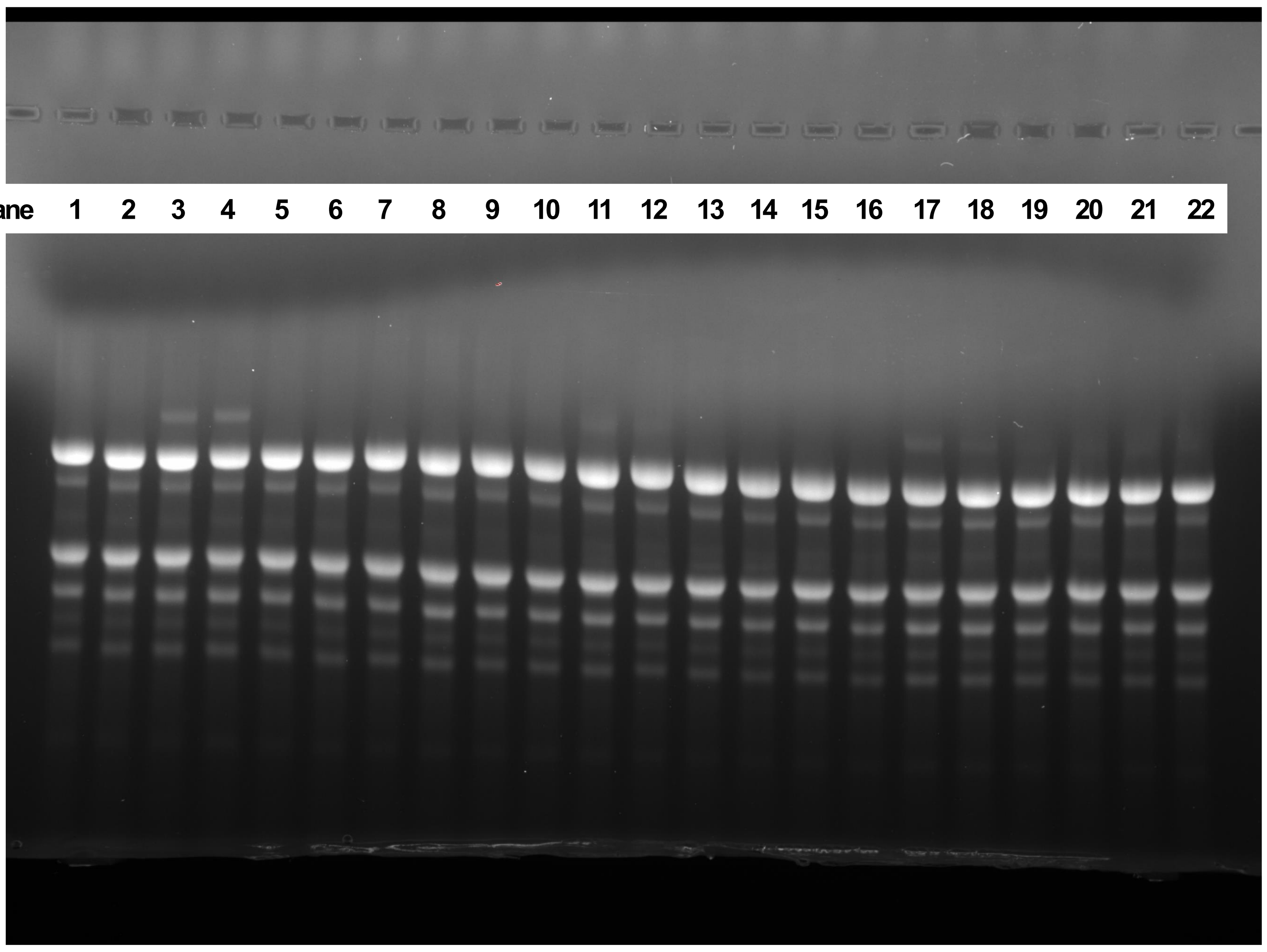

Loading order:
<u>Figure 4B</u>

1: 7dpai SL (wt N.b.) Mock
2: 7dpai SL (wt N.b.) Mock
3: 7dpai SL (wt N.b.) wt TCV
4: 7dpai SL (wt N.b.) wt TCV
5: 7dpai SL (wt N.b.) K389E
6: 7dpai SL (wt N.b.) K389E
7: 7dpai SL (wt N.b.) E392K
8: 7dpai SL (wt N.b.) E392K
9: 7dpai SL (wt N.b.) KEEK
10: 7dpai SL (wt N.b.) KEEK
11: 7dpai SL (wt N.b.) W753L
12: 7dpai SL (wt N.b.) W753L
13: 7dpai SL (wt N.b.) A113V
14: 7dpai SL (wt N.b.) A113V
15: 7dpai SL (wt N.b.) A113V + K389E
16: 7dpai SL (wt N.b.) A113V + K389E
17: 7dpai SL (wt N.b.) A113V + E392K
18: 7dpai SL (wt N.b.) A113V + E392K
19: 7dpai SL (wt N.b.) A113V + KEEK
20: 7dpai SL (wt N.b.) A113V + KEEK
21: 7dpai SL (wt N.b.) A113V + W753L
22: 7dpai SL (wt N.b.) A113V + W753L

Perdoncini Carvalho et al 2026

Original Bot ang Gel for Figure 4D

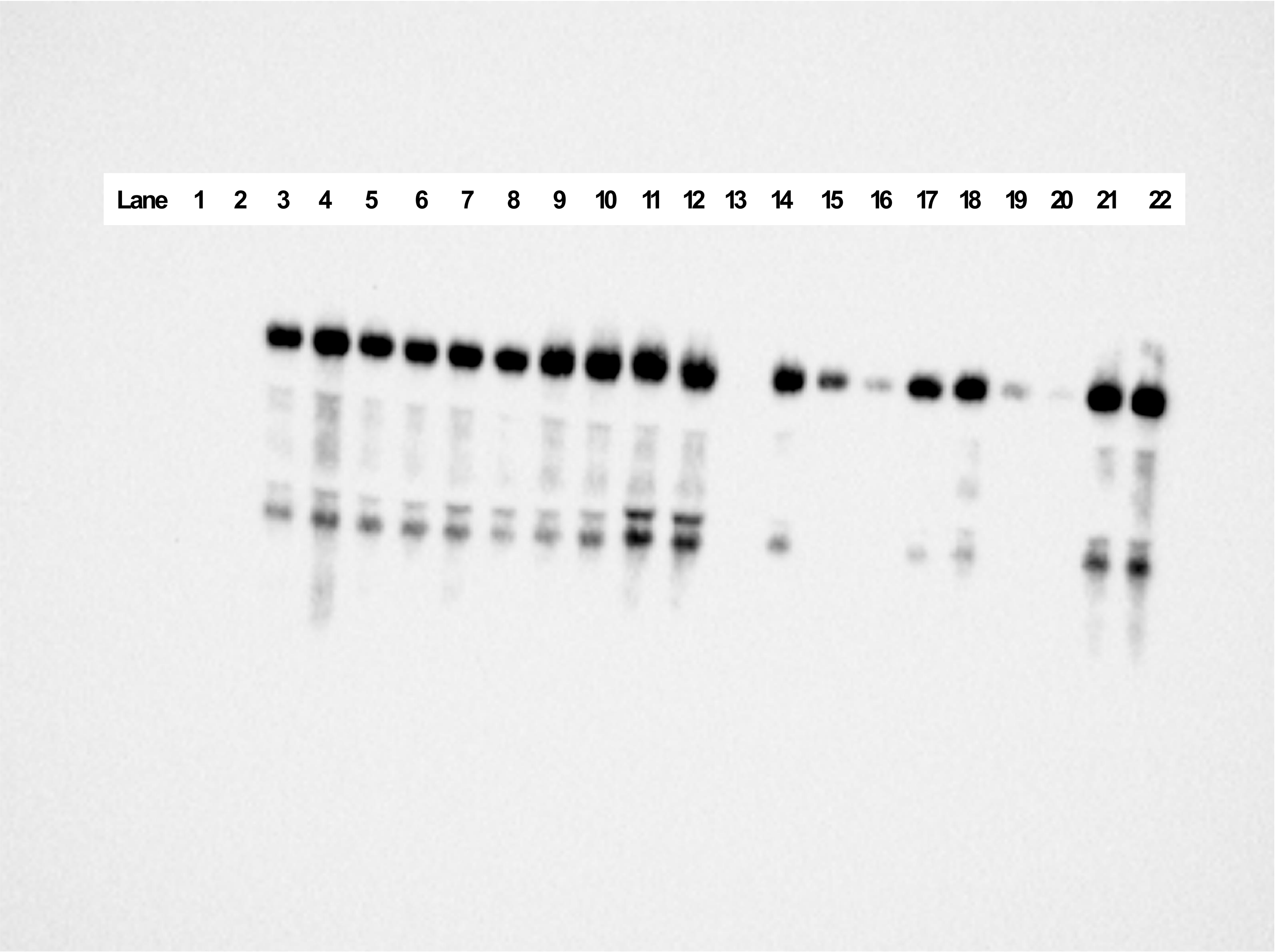

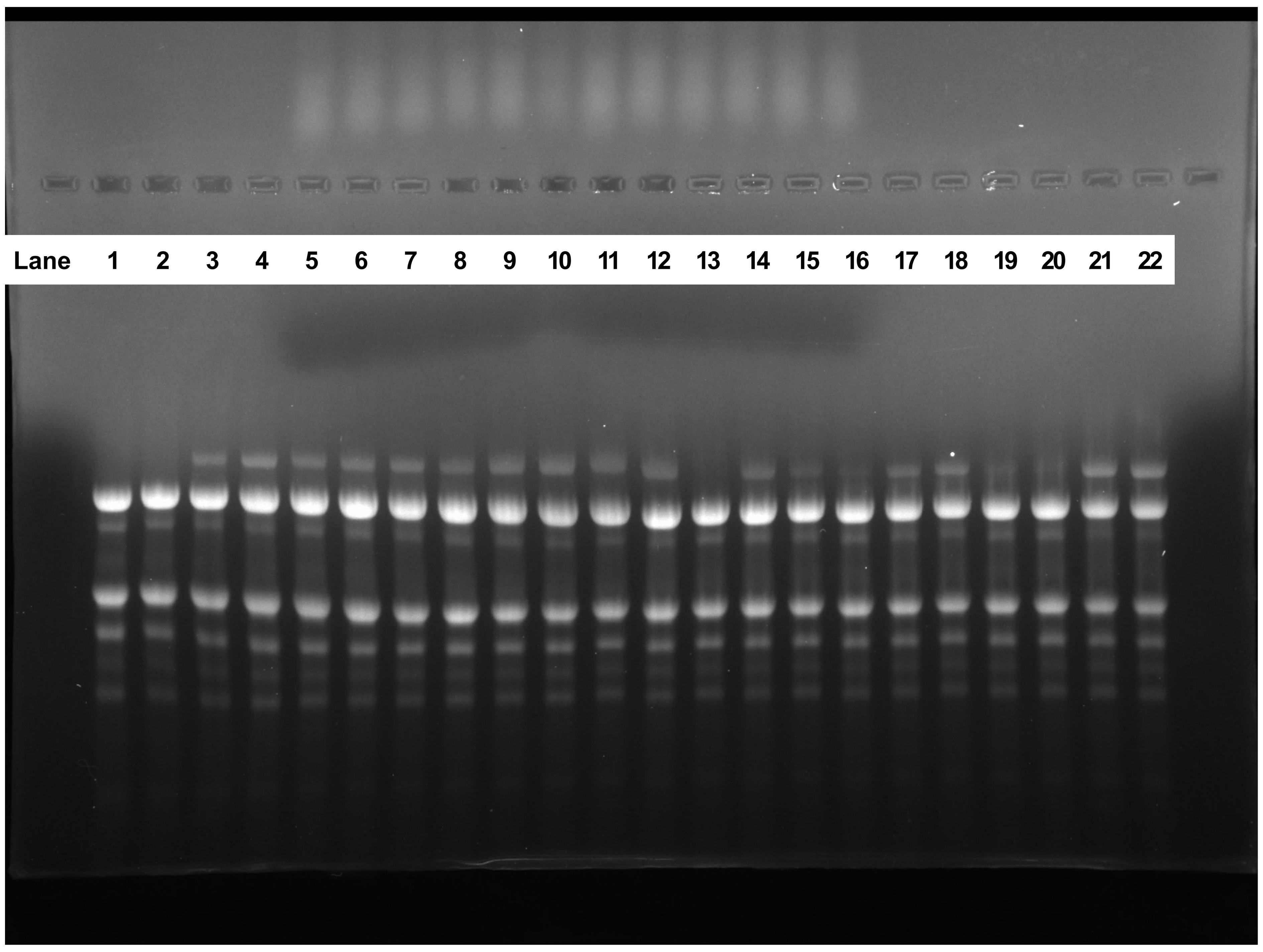

Loading order:
<u>Figure 4B</u>
1: 14dpai SL (wt N.b.) Mock
2: 14dpai SL (wt N.b.) Mock
3: 14dpai SL (wt N.b.) wt TCV
4: 14dpai SL (wt N.b.) wt TCV
5: 14dpai SL (wt N.b.) K389E
6: 14dpai SL (wt N.b.) K389E
7: 14dpai SL (wt N.b.) E392K
8: 14dpai SL (wt N.b.) E392K
9: 14dpai SL (wt N.b.) KEEK
10: 14dpai SL (wt N.b.) KEEK
11: 14dpai SL (wt N.b.) W753L
12: 14dpai SL (wt N.b.) W753L
13: 14dpai SL (wt N.b.) A113V
14: 14dpai SL (wt N.b.) A113V
15: 14dpai SL (wt N.b.) A113V + K389E
16: 14dpai SL (wt N.b.) A113V + K389E
17: 14dpai SL (wt N.b.) A113V + E392K
18: 14dpai SL (wt N.b.) A113V + E392K
19: 14dpai SL (wt N.b.) A113V + KEEK
20: 14dpai SL (wt N.b.) A113V + KEEK
21: 14dpai SL (wt N.b.) A113V + W753L
22: 14dpai SL (wt N.b.) A113V + W753L

Lanes marked with “X” are not part of the Figure. Same for other blots and gel images.

Perdoncini Carvalho et al 2026

Original Gel for Figure 5A

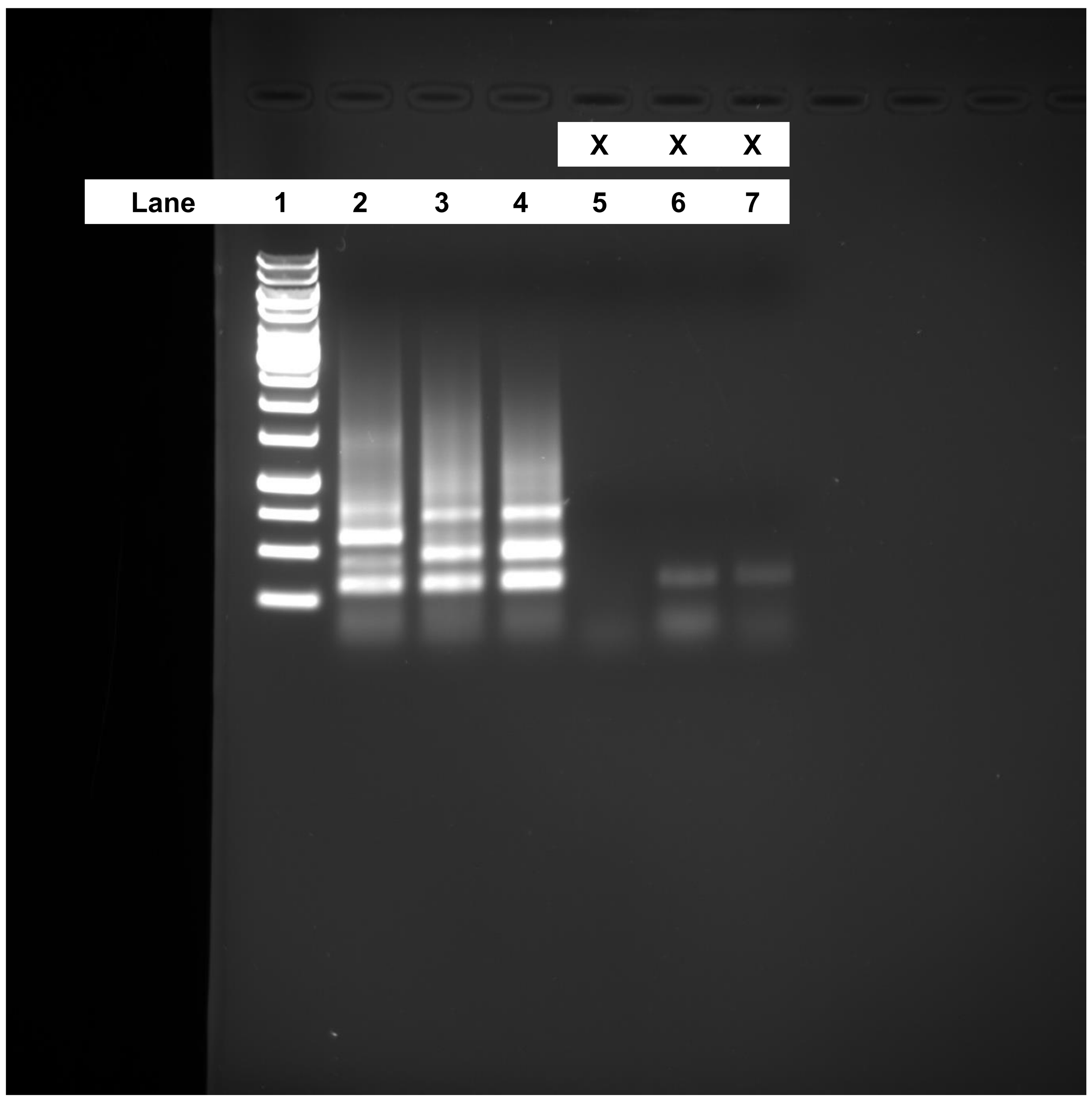

Lanes marked with “X” are not part of the Figure. Same for other blots and gel images.

Loading order:
Figure 5A
1: GeneRuler 1 kb DNA Ladder (Thermo)
2: RT-PCR - wt TCV (4dpai IL)
3: RT-PCR - KEEK (4dpai IL)
4: RT-PCR - A113V-W753L (4dpai IL)
