# Supplementary material for: Naturally occurring mutations in replication proteins of a small RNA virus that alter the number, sizes, and relative abundances of subgenomic RNAs
Source: PLoS Pathog. 2026 Jul 7;22(7):e1013842. doi: 10.1371/journal.ppat.1013842 (PMC13340797; doi:10.1371/journal.ppat.1013842)
Supplement: S1 Text — (DOCX) [file ppat.1013842.s004.docx]

**SUPPLEMENTARY FILES**

**Naturally occurring mutations in replication proteins of a small RNA virus that alter the number, sizes, and relative abundances of subgenomic RNAs**

Camila Perdoncini Carvalho^1^, Deya Wang^1,2^, Junping Han^1^, Khwannarin Khemsom^1^, Hanqiao Chen^3^, Yizhi Jane Tao^3^, Feng Qu^1,^ *

^1^Department of Plant Pathology, The Ohio State University, Wooster, OH 44691

^2^ZaoZhuang University, Zaozhuang, Shandong, China

^3^Department of BioSciences, Rice University, Houston, TX 77251

*Correspondence: [qu.28@osu.edu](mailto:qu.28@osu.edu)

**The SI files include**:

- S1 Text – detailed Materials and Methods

**S1 Text**

**Material and Methods**

**Constructs**. TCV-dMP_sg2R was modified from TCV_sg2R [1,2]. TCV_sg2R is a shuttle plasmid capable of replicating in both *Escherichia coli* and *Agrobacterium tumefaciens* (strain C58C1), and it harbors a TCV cDNA engineered to express the mCherry (denoted as R for the red fluorescence emitted by mCherry) reporter protein from TCV sgRNA2 (sg2). The TCV_sg2R cDNA is preceded by the 2X35S promoter so that the primary, replication-launching transcripts are synthesized by the DNA-dependent RNA polymerase II (Pol II) of the host cells [3]. TCV-dMP_sg2R contains an additional modification that deletes positions 2,424-2,517 of TCV cDNA, abolishing the production of both p8 and p9 MPs [4]. The wt TCV construct was based on the same vector plasmid, but has the full-length, unmodified TCV cDNA as the insert [5]. All four Mut17 mutations were introduced into TCV-dMP_sg2R by digesting plasmids with XmaJI (AvrII) and BamHI and ligating the mutation-containing fragments with T4 DNA ligase (Thermo Fisher Scientific). To create mutants with single mutations, fragments containing mutations C401U (A113V), C972T (N303N), and G2332T (G757*) were PCR-amplified using appropriately designed primers, and introduced into both TCV-dMP_sg2R and wt TCV between the XmaJI and NruI (Bsp68I), NruI and ApaI, and SalI and BamHI sites, respectively, using Gibson Assembly (NEBuilder HiFi DNA Assembly, New England BioLabs). The G674A (R204H) mutation was generated through PCR-based mutagenesis and introduced between the XmaJI and NruI sites. The A113V second-site mutations A1228G (K389E), G1237A (E392K) and KEEK (the combination of both K389E and E392K) were introduced between the NruI and ApaI sites, and the mutation G2321T (W753L) was ligated between SalI and AatII sites. All four mutations were introduced by site-directed mutagenesis, and cloning was performed by 3-party Gibson Assembly. Primer sequences are available upon request. All inserts were sequenced to make sure no errors were introduced by PCR.

**Plant Materials.** Both wild type (non-transgenic) and MP+ (transgenic, expressing RCNMV MP) *N. benthamiana* plants [6,7] were grown in a greenhouse. After infiltration, plants were kept in growth chamber, with a 16-h day length, and temperatures of 25 °C.

**Agrobacterium infiltration (agro-infiltration).** Constructs were transformed into electrocompetent *Agrobacterium tumefaciens* strain C58C1 as described previously [1]. Fresh Agrobacterium suspensions carrying the various binary constructs were pelleted and resuspended in agroinfiltration buffer (10 mM MgCl2, 10 mM MES, and 100 µM acetosyringone) to OD_600_ = 1. After 3 hours of incubation at room temperature, suspensions were diluted to final OD_600_ of 1, 0.1, or 0.01. Agrobacteria harboring constructs that do not encode the canonical silencing suppressor (TCV p38) were mixed 1:1 with those harboring a TBSV p19-expressing construct to alleviate RNA silencing-mediated RNA degradation [2,7]. Suspensions were infiltrated into the first two true leaves of young *Nicotiana bethamiana* plants via a small wound, using a 3 ml needleless syringe.

**RNA extraction and Northern blotting**. Total RNA was extracted from agro-infiltrated *N. benthamiana* leaves 4 days post agro-infiltration (dpai), and from systemic leaves at 7-, 14-, 21- and 42-dpai using the Direct-zol RNA Miniprep kit (Zymo Research). All extractions included a DNase treatment to remove DNA contamination. RNA was quantified with NanoDrop and Northern blotting was performed as described [2,8], with small modifications. Namely, 4 μl of a 10 mM mix of three TCV specific probes tagged with a 5’ biotin label was added to 5 ml of hybridization buffer (PerfectHyb Plus Hybridization Buffer, Sigma-Aldrich) and kept rotating overnight at 46^o^C, following detection with the Chemiluminescent Nucleic Acid Detection Module (Thermo Fisher Scientific). Image blots were acquired using the Chemi setting in a ChemiDoc MP Imaging System (Bio-Rad).

**RT-PCR and Sequencing**. Total RNA isolated from systemic leaves of individual plants at 14-, 21- or 42-dpai were subjected to 2-step RT-PCR to identify the prevalent TCV variant in apical tissues. RT was performed with the primer TCV-2634R, using the RevertAid Reverse Transcriptase (Thermo Fisher Scientific). Following PCR was carried out with the primers Tg3-TCV-25F and Tg4-TCV-2425R, both at 500 nM, utilizing the Phusion High Fidelity Master Mix (Thermo Fisher Scientific). Positive PCR samples that amplified a 2,450-bp cDNA fragment were column purified and sent for Sanger sequencing with three overlapping primers (TCV-946R, TCV-832F, and TCV-1627F), covering the entire TCV p88 coding sequence.

**Mapping of sgRNAs 5’ and 3’ ends.** Total RNA extracted from infiltrated leaves at 4-dpai were selected for 2-step RT-PCR. RT was performed with the primer Tg3-TCV-3802F, and PCR with the divergent primers TCV-3872F and TCV-2741R (Fig. 5C), amplifying both the 3’ and 5’ ends of sgRNAs. Positive PCR results (Fig. 5A) were column purified and cloned into the pJET1.2/blunt cloning vector (Thermo Fisher Scientific). Single colonies were selected, cultured overnight, and their plasmids extracted with ZR Plasmid Miniprep (Zymo Research). Plasmids with different size inserts were sent for Sanger sequencing with the pJET1.2 Reverse Sequencing Primer.

The 5’ termini TCV sgRNAs were additionally mapped with a 5’ RACE approach [9]. Briefly, a RACE Adaptor primer with sequence 5’-GGCCTCTCACCACCAAAGAAGCAGTCAAGTCTTACCGAAC was ligated to the 5’ ends of sgRNAs (as well as gRNA and other 5’ uncapped cellular RNAs) using T4 RNA ligase 1 (New England Biolabs). The resulting RNA samples were then subjected to reverse transcription with the primer TCV-2965R (5’-CCGAGGCTGGGTGGAAACCT) to produce sgRNA-specific cDNAs. The cDNAs were then PCR-amplified with TCV-2965R and another RACE PCR primer (5’-AAGCAGTCAAGTCTTACCGAAC). The amplified PCR products were subsequently gel-purified and cloned into pJET1.2 (Thermo Fisher Scientific). Plasmid clones with inserts of expected sizes were subjected to whole plasmid sequencing (QUINTARA Bioscicence) to resolve the sequence identities of inserts.

**Supplementary References**

1. Perdoncini Carvalho, Han J, Khemsom K, Ren R, Camargo LEA, Miyashita S, et al. Single-cell mutation rate of turnip crinkle virus (-)-strand replication intermediates. PLOS Pathog. 2023;19: e1011395. doi:10.1371/journal.ppat.1011395

2. Zhang S, Sun R, Perdoncini Carvalho, C., Han J, Zheng, L., Qu, F. Replication-Dependent Biogenesis of Turnip Crinkle Virus Long Noncoding RNAs. J Virol. 2021;95: 10.1128/jvi.00169-21. doi:10.1128/jvi.00169-21

3. Zhang X-F, Sun R, Guo Q, Zhang S, Meulia T, Halfmann R, et al. A self-perpetuating repressive state of a viral replication protein blocks superinfection by the same virus. PLOS Pathogens. 2017;13: e1006253. doi:10.1371/journal.ppat.1006253

4. Li W, Qu F, Morris TJ. Cell-to-Cell Movement of Turnip Crinkle Virus Is Controlled by Two Small Open Reading Frames That Functionin trans. Virology. 1998;244: 405–416. doi:10.1006/viro.1998.9125

5. Qu F, Ren T, Morris TJ. The Coat Protein of Turnip Crinkle Virus Suppresses Posttranscriptional Gene Silencing at an Early Initiation Step. Journal of Virology. 2003;77: 511–522. doi:10.1128/jvi.77.1.511-522.2003

6. Vaewhongs AA, Lommel SA. Virion Formation Is Required for the Long-Distance Movement of Red Clover Necrotic Mosaic Virus in Movement Protein Transgenic Plants. Virology. 1995;212: 607–613. doi:10.1006/viro.1995.1518

7. Qu F, Morris TJ. Efficient Infection of Nicotiana benthamiana by Tomato bushy stunt virus Is Facilitated by the Coat Protein and Maintained by p19 Through Suppression of Gene Silencing. MPMI. 2002;15: 193–202. doi:10.1094/MPMI.2002.15.3.193

8. Sun R, Han J, Zheng L, Qu F. The AC2 Protein of a Bipartite Geminivirus Stimulates the Transcription of the BV1 Gene through Abscisic Acid Responsive Promoter Elements. Viruses. 2020;12. doi:10.3390/v12121403

9. Chkuaseli T, White KA. Dimerization of an umbravirus RNA genome activates subgenomic mRNA transcription. Nucl Acids Res. 2023;51: 8787–8804. doi:10.1093/nar/gkad550
